# The Impact of Intimate Partner Violence on Homelessness and Returns to Housing: A Qualitative Analysis From the California Statewide Study of People Experiencing Homelessness

**DOI:** 10.1177/08862605241259006

**Published:** 2024-07-15

**Authors:** Anita S. Hargrave, Kelly R. Knight, Zena K. Dhatt, Grace Taylor, Dez Martinez, Margot Kushel

**Affiliations:** 1 University of California San Francisco (UCSF), CA, USA; 2 San Francisco VA Health Care System, CA, USA; 3 UCSF Center for Vulnerable Populations, CA, USA

**Keywords:** domestic violence, anything related to domestic violence, intervention/treatment, domestic violence and cultural contexts

## Abstract

Homelessness is a public health concern in California and throughout the United States. Intimate partner violence (IPV) is a risk factor for experiencing homelessness. Few studies have examined the interplay between IPV, homelessness, and housing. Qualitative methods can provide a greater understanding of the lived experience of IPV and homelessness to identify potential solutions. We purposefully sampled 104 adults who reported experiencing IPV in the California Statewide Study of People Experiencing Homelessness (CASPEH), a representative, mixed-methods study. We administered semi-structured interviews focusing on IPV and six other topic areas pertaining to homelessness from October 2021 to May 2022. We created and applied a codebook with a multidisciplinary team using a hybrid of deductive and inductive logic. Our analysis included all participants who discussed IPV and homelessness across the seven studies. We conducted a thematic analysis using an interpretivist approach and informed by grounded theory. We found that violence within a partnership was multidimensional (physical, sexual, emotional, and financial) and bidirectional. We identified six themes: (1) IPV precipitated and prolonged homelessness; (2) Need for housing, financial stability, and material resources influenced staying in abusive relationships; (3) Alcohol and illicit substance use exacerbated violence between partners; (4) Participants struggled to find resources in domestic violence (DV) shelters; (5) The healthcare system did not provide substantial support; and (6) discrimination and stigma influenced equitable access to housing and DV resources. Experiencing IPV contributed to homelessness and impeded returns to housing. Limitations in current IPV resources impede care. We propose equitable expansion of survivor-centered services that improve access to long-term subsidized housing, prevent IPV and homelessness with flexible funding options, and facilitate rapid exits from homelessness through trauma-informed, non-congregate shelter that transitions to permanent housing.

## Introduction

Intimate partner violence (IPV) affects more than 15 million people in the United States each year (Leemis et al., 2022). Also known as *domestic violence*, IPV refers to physical, sexual, or psychological violence or stalking by an intimate partner (Breiding et al., 2015; Leemis et al., 2022). IPV can have negative health outcomes, including physical injury, depression, post-traumatic stress disorder, and death (Breiding et al., 2015; Leemis et al., 2022). IPV is closely associated with unstable housing and homelessness, which compound these negative health consequences (Chan et al., 2021; Daoud et al., 2016; Jasinski et al., 2005; A. E.Montgomery et al., 2018; Pavao et al., 2007). California is home to 171,521 people experiencing homelessness in the United States nightly, which includes more than 12,500 survivors of IPV (U.S. Dept. of Housing and Urban Development, 2022).

IPV is rooted in power, control, and conflict (Jewkes, 2002). Coercive dynamics and violence cause barriers to obtaining emergency shelter and long-term housing for survivors of IPV (Chan et al., 2021; Fraga Rizo et al., 2022). Controlling behaviors (i.e., impeding access to financial independence or social networks) increase the risk of homelessness (Adams et al., 2012; Bassuk, 1996; World Health Organization [WHO], 2012). Survivors require a high level of confidentiality when leaving abusive relationships to avoid retaliation (Decker et al., 2022). These barriers to housing are amplified for people with historically marginalized identities who experience the intersecting effects of structural and interpersonal discrimination (WHO, 2012).

To inform efforts to address homelessness among IPV survivors, we used interpretivist methodologies to understand how IPV intersects with homelessness and returns to housing (Duke et al., 2023; Martin & Kunnen, 2008). Extant qualitative literature exploring experiences of homelessness among IPV survivors has mostly been conducted among women (Rempel et al., 2024) of reproductive age (Clough et al., 2014; Kulkarni & Notario, 2023; Tucker et al., 2005) and accessing services for homelessness (Clough et al., 2014; Fraga Rizo et al., 2022; Gezinski & Gonzalez-Pons, 2021; Tucker et al., 2005; Wood, Schrag, et al., 2022). Few have integrated the experiences of men (Fraga Rizo et al., 2022; Kulkarni & Notario, 2023), people 50 years and older (Clough et al., 2014; B.Yu et al., 2020), or who are unsheltered. The homeless population is changing in ways that necessitate ongoing analyses of how it is impacted by IPV—it is aging, includes more unsheltered homelessness, and is increasingly impacting the Latinx community (Brown et al., 2016; Tobias, 2022).

Our study included qualitative interviews conducted in conjunction with a representative survey of adults experiencing homelessness in California. We address gaps in current literature by studying contemporary experiences of IPV and homelessness among a cohort of homeless women *and* men, including those who are unsheltered and not accessing services for homelessness. We examined the range of IPV experiences, their impact on homelessness, and experiences with existing programs or systems to support survivors. The study aims to be instructive for homelessness and housing programs and policies.

## Methods

From October 2021 to May 2022, we conducted the California Statewide Study of People Experiencing Homelessness (CASPEH). This representative, mixed-methods study included adults who were experiencing homelessness in accordance with the federal Homeless Emergency Assistance and Rapid Transitions to Housing (HEARTH) Act (Housing and Urban Development Department, 2010). Detailed findings and methods are published separately (Kushel et al., 2023; Duke et al., 2023).

### Recruitment and Sample Selection

To enroll the 3,200 survey participants, we selected participants in eight counties representing eight diverse regions and populations within the state (Central Coast and Southern California; Inner Bay Area; Inland California; Los Angeles; Northern California; Northern Central Valley; Outer Bay Area; and Southern Central Valley) (Duke et al., 2023; Kushel et al., 2023). We recruited from a sample of all venues where adults experiencing homeless congregate, including shelters, community centers, food programs, showers, as well as unsheltered encampments (e.g., vehicles, tents, makeshift structures, or outside) (Burnam & Koegel, 1988). We randomly selected venues proportional to their size. Within each venue, we randomly sampled participants. We supplemented with respondent-driven sampling for hard-to-reach populations, including survivors of domestic violence (DV) (Heckathorn, 1997; Raifman et al., 2022). Staff recruited a subset of 365 of these participants in real time to complete one of seven qualitative interviews, depending on their answers to the survey questions, immediately after completing the structured interview. Participants completed the in-depth interview within an hour of recruitment. Each qualitative study had a different primary focus as it pertained to homelessness: (1) IPV, (2) incarceration, (3) Latino/x experiences, (4) Black experiences, (5) precipitants, (6) behavioral health, and (7) barriers to returns to housing (Duke et al., 2023). In this study, we analyzed qualitative data from participants (*N* = 104) in any of the seven qualitative sub-studies who discussed IPV.

### Data Collection

Interviews averaged 30 to 45 min, and were audio-recorded, transcribed, and translated verbatim. Trained interviewers conducted in-depth interviews on the same day and at the same site as the paired structured interview (Duke et al., 2023). We continued data collection until thematic saturation. Participants received a $30 gift card for questionnaires and $30 for qualitative interviews. The University of California, San Francisco IRB approved this study. All participants provided informed consent.

### Data Analysis

Consistent with grounded theory approaches, we started data analysis with initiation of the data collection and captured emergent themes using thematic memos ( P.Montgomery & Bailey, 2007). We used deductive and inductive logic for code development and thematic analysis to identify themes (Attride-Stirling, 2001; Pope & Mays, 2020). Two researchers coded each transcript using Dedoose and compared coding regularly to reach intercoder reliability and consensus (SocioCultural Research Consultants, LLC, 2021).

For this manuscript, we conducted a thematic analysis focused on excerpts coded for “IPV/DV” with the aim of understanding the intersection of IPV and homelessness among participants. We used an interpretivist approach, which derives meaning from the perspectives of those who lived through the phenomenon under study (Duke et al., 2023; Pope & Mays, 2020). Therefore, we regularly met with a multidisciplinary team composed of healthcare providers, qualitative researchers, and people with lived experience of homelessness and IPV to analyze the data together, focusing on understanding our participants’ perspectives on IPV and its connection to their housing status as well as our positionality in the interpretation.

## Results

Among the 365 participants who completed a qualitative interview, 104 discussed IPV and are included in this study. Of those, 49 were enrolled in the IPV sub-study. A majority of the 104 participants were 25 to 49 years old (56%) and identified as cis-gender women (75%). Approximately 30% of participants identified as Black, 30% as white, and 24% as Latinx/Hispanic. At the time of the interview, 14% of the participants were staying in a DV shelter (Table 1).

**Table 1.** Participant Characteristics and Experiences.

Participant sociodemographic data (*N* = 104)	*N* (%)
Age (in years)	
18–24	2 (2)
25–49	58 (56)
50+	44 (42)
Race/Ethnicity	
Asian American/Pacific Islander	1 (1)
Black	31 (30)
Latinx/Hispanic	25 (24)
Native American/Alaskan/Indigenous	4 (4)
Other/Multiracial	12 (12)
White	31 (30)
Gender	
Cis-man	24 (23)
Cis-women	78 (75)
Transgender	2 (2)
Location	
Domestic violence shelter	15 (14)
Non-domestic violence shelter	89 (86)
Interview subtype	
Intimate partner violence and homelessness	49 (47)
All other sub-studies	55 (53)

Violence within partnerships was complex and multidimensional, including physical, sexual, psychological, and financial abuse. IPV could be bidirectional, with the same person being a perpetrator and target of violence. Although both men and women reported being harmed by IPV, women described a higher burden of severe physical and sexual violence. We identified six principal themes: (1) IPV precipitated and prolonged homelessness, (2) the need for housing, financial stability, and material resources influenced staying in harmful relationships, (3) alcohol and illicit substance use exacerbated violence between partners, (4) participants struggled to access supportive services for IPV or find permanent housing in the DV shelter system, (5) the healthcare system did not provide substantial support, and (6) discrimination and stigma influenced equitable access to housing and DV resources.

### Precipitating and Prolonging Homelessness

Experiences of IPV contributed to or led directly to initial and ongoing homelessness. Participants were forced to exit housing due to IPV. Violence could lead to property damage and noise complaints, precipitating orders to vacate. Property owners expressed displeasure with survivors returning to partners who had caused property damage: “The main owner kicked me out . . . they’re like, ‘Why are you getting back with him? He just broke the whole garage door, and I have to pay $500 to fix it’” (50-year-old woman). Some participants received formal evictions; others agreed to be releases from the lease to avoid having an eviction record. A participant lost the housing that her family had offered her in exchange for work because of IPV.

For six months they were going to pay for [rent] as long as we could take the business . . . He was fighting with me so much they just said, “No, we’re not going to do it because you guys can’t handle this. You guys are fighting too much.” (38-year-old woman)

Some participants became homeless after they were forced out of the home by their partner. They reported that they were forced to leave to avoid harm and escalating abuse:“I’m here now [homeless] because I just left the house. I just needed to leave because it was more physical and more violent” (45-year-old woman). Partners used violence and intimidation to prevent participants from using the legal system or advocating for their ability to stay in their current housing. Many partners were homeowners or had their name on the lease or the housing subsidy, granting them the right to remain in the house and leaving the participant without the choice to do so.

I tried calling [the police]. But he played on their side because he’s the homeowner . . . [The officer] said, “Well, you have until 4:00 in the morning to vacate this place.” I said, “This is my home. I’ve been living here for three years” and [the officer] was like, “I don’t care.” (56-year-old woman)

Once homeless, participants reported changing locations (including unsheltered areas and temporary motels) due to IPV. IPV interfered with returns to housing by leaving survivors with poor credit or eviction records: “He got us kicked out of a place. It was the fighting . . . that was the only time we got evicted. Made it harder to get [housing] with the eviction on the credit” (40-year-old woman). A woman explained that her partner stalked her, thus ensuring that she was evicted from several homes. The violence destroyed her identification and phone. Given her limited financial resources, she had trouble replacing them to access IPV services.

He would break windows just do whatever means necessary to get me out of the house . . . [a] couple houses actually. He took my phone and [my ID] got burned up in the fire. I’m trying to get the ID so I can get the free phone. But it’s hard even to eat out here, let alone have the extra money to get my ID. (40-year-old woman)

### Staying in an Abusive Relationship for Housing, Stability, and Material Resources

Participants stayed in harmful relationships to protect their housing or preserve resources. Financial control and coercion were a prominent feature of IPV among our participants. Given the lack of financial independence, many feared losing their ability to sustain themselves or their family without their partner: “In my opinion, probably ninety percent of the people that are in abusive relationships, would get out if they had an out to go to, if they weren’t going to lose their kids, their home, their financial ability to function day to day, then it would be a whole lot easier to leave” (43-year-old woman). The lack of financial independence was compounded by uncertainty regarding supportive services for IPV survivors:

Every time when you ask for something, [case managers] tell you, “It’s not guaranteed but we will work on it.” So, you get the feeling of scared. If it’s not guaranteed and not going to happen, then what will happen? So let me stay with my husband. At least I have a place. (37-year-old woman)

Participants reported staying in relationships to save money, obtain resources, create a safety plan, and avoid exposing their children to homelessness: “Even when we were still together . . . I got on a list [for housing] because I knew I was going to break up with him and I knew I couldn’t afford it on my own. I preplanned the whole thing. I took a long time to get him out of the picture. I didn’t want to be out on the street with my kid, with my baby, you know?” (42-year-old woman) They feared that if they were to become homeless, their children would be removed by Child Protective Services (CPS).

Especially when you have kids, it’s scary. I’d be hearing stories about the CPS. Once they know there is a domestic violence happening, they’re going to take all of your kids from you. (37-year-old woman)

### Role of Alcohol and Substances in Exacerbating Violence Between Partners

Alcohol and substance use contributed to escalating violence and difficulty maintaining housing. Partners’ alcohol use led to “blacking out,” which made them more violent: “He’d drink two Long Islands. Doesn’t remember anything and peed the bed and blamed it on me and then beat me” (22-year-old woman). When partners could not obtain more of the substance or experienced withdrawal, they became more violent: “When he didn’t have money to buy his fentanyl, he’d be abusive and be angry with me . . . he used to be like, ‘You’d better find a way for me to get it’” (50-year-old woman). Substance use by participants and their partners could trigger past trauma. It was connected to feelings of guilt, desperation, lack of control, and financial strain, as well as insomnia and irritability, which would heighten conflict between partners.

He’ll get too many drinks and he’ll get to think about his past [trauma]. And then, I’m the only person there. So, naturally, I’m the outlet. It was just like a vicious cycle, no matter how much you try to be sweet, loving, kind. I think the comedown and trying to kick it was the worst thing from him. Because that was a lot of arguments, the guilt from doing it. He was doing formaldehyde. If you’re smoking embalming fluid, you’re going to be crazy. And it’s you’re cranky, you’re mad because you can’t sleep. But you got it in your system, so you still want to be up. (45-year-old woman).

Participants identified that use of methamphetamines increased violent behavior in their partnerships: “There was no domestic violence, without the amphetamine” (53-year-old man). Another participant recounted that amphetamine intoxication led him to feel out of control and behave violently:“[Meth] would make me lose my mind . . . I would start yelling out like a demon. And I would be like, ‘Man, I got to stop this.’ Even though I wanted to stop, I couldn’t stop acting like it. So, it kind of scared me. We would get violent; it was the drugs” (34-year-old man). The violent behavior could directly contribute to housing loss: “[My partner] thought I was getting too aggressive and kicked me out of the house . . . I was abusing pills at the time, which was making me aggressive. I would just have kinda mental breakdowns throughout the day” (28-year-old woman).

### Difficulty Finding Resources in DV Shelters

Participants noted needing long-term or permanent subsidies due to the financial instability created by being in an abusive relationship. DV shelters faced systemic challenges, such as limited budget and staffing, rendering them unable to fully meet survivor needs. Participants wished for improved access to permanent affordable housing and services for survivors: “I was hoping that [DV shelters] had access to more information than the general public, or services that could expedite more quickly some type of permanent housing or help with low income, and those things just aren’t provided, as well as transportation to or from any appointment” (43-year-old woman). They noted that flexible financial support could help with additional needs for transportation, food, childcare, and relocation costs: “There should be more things for women that are abused . . .[like] grants, some type of funds to help them get on their feet” (56-year-old woman).

Participants desired more case managers within DV shelters to help navigate housing and social or health services because they found it difficult to advocate for themselves while working through trauma resulting from IPV: “So for these [IPV] situations it seems like you have to stay on top of the people that are supposed to be helping you to get the help that you need, and that’s really hard. When you’re going through trauma, it’s hard to even get up sometimes, you feel really low” (35-year-old woman). Participants worried that the time limitations on stays in DV shelters would not be sufficient to return to stable housing.

They make you sign something saying that you’re not promised housing after you leave here. We’re not safe after we leave here because we’re going to go back in the street, where our abusers are at . . . What happens after 60 days if I don’t find [housing]? There’s no bridges here. I’m scared (50-year-old woman).

Some participants voiced appreciation for the aid that they received in DV shelters. They were grateful for private rooms, friendly staff, and safety measures: “They’re very helpful with anything you need. The people are nice, friendly . . . I’m not in the big part no more with everybody. I have my own little cottage [in the shelter] . . . And I feel safe. It’s gated” (39-year-old woman). However, many participants discussed their inability to access DV shelters due to lack of available beds: “I called [the shelter], they said that they didn’t have any room available. It seemed to me the severity of my situation was not deemed an emergency” (43-year-old woman). Barriers to accessing DV shelters including needing to provide proof of IPV through police or hospital reports: “I was told I needed to have my proof that I went through [IPV] pretty much, like police report and or hospital things. I can’t prove any of that. I didn’t go to the hospital because I didn’t want to go and get in trouble” (22-year-old woman). Other barriers included not having transportation to the DV shelter and not being allowed to bring children or pets: “I had to experience this homelessness, and it came from domestic violence . . . you’re calling [DV shelters] and giving reports and there’s no help for victims . . . there are no emergency shelters for families [with children], not even for DV victims” (44-year-old-woman).

### Participants Reported Not Receiving Support in The Healthcare System and Did Not View It As a Potential Source of Help

We found that for most participants, the healthcare system was not a source of IPV support services or housing resources. Many reported not disclosing IPV to healthcare clinicians because they did not believe clinicians had resources or knowledge to support them. A woman explained: “I did [talk to a clinician], but there wasn’t really any assistance at the time. It was like, ‘Oh, I’m sorry [about the IPV]. And here’s your medicine . . . I think that if there was more proactive assistance when you’re facing domestic violence, things have a better outcome’” (43-year-old woman).

Participants stressed the importance of supporting survivors without judgment: “I came for help. I didn’t come for you to judge me. I make mistakes, but I need the help” (35 year-old-woman). They thought that healthcare providers could play a greater role in supporting survivors, particularly for safety planning, mental health treatment, and expediting entrance into shelter.

[Healthcare providers] can do more to protect the person that was attacked. They should be able to call a DV safe house and say, “Hey, we have this client that just came. She was assaulted by her boyfriend. Could you send an advocate down her to speak with her?” It would’ve helped a whole lot if they would’ve gone, “Hey, look, even if you do decide to go back, we’re going to try to help you right now, so that we can at least encourage you not to go back” (35-year-old woman).

Participants voiced a lack of trust in the healthcare system (“I don’t trust doctors a lot” 55-year-old woman.) and concern that seeking care would lead to involvement in the criminal legal system. A woman explained that when she went to the hospital, she gave clinicians a false excuse for her IPV-related injuries to avoid police involvement; however, they made the report without her consent, which escalated violence in the partnership: “I went to the hospital. They didn’t believe that I was jumped again. They called [the police] without my knowledge . . . and he tried to kill me again” (37-year-old woman). Participants described negative experiences with healthcare clinicians. Some reported that healthcare providers were insensitive to their individual experience: “[Clinicians] have to go through everybody and hear everybody. To them, everything is the same. But see, all domestic violence is different” (38-year-old woman). A participant was falsely told she had to talk to the police to receive medical attention: “First [the doctors] sent the police in. And I said, ‘Why are they here?’ And they said, ‘Well, we wanted to ask you some questions. If you refuse—you’re going to have to refuse medical attention’” (50-year-old woman).

### Discrimination, Stigma, and Equitable Access to Housing and DV Resources

Participants expressed concern that there were discriminatory practices in access to housing, emergency shelter, and DV services. Black and Latinx participants recounted feeling that they were denied access to shelter because of their race or ethnicity. A Latina participant noted that she believed there was the preferential treatment of white survivors who sought to access DV shelters through the healthcare system: “[Doctors] should ask me if I need a safe place, and if I need to go to DV shelter right away. Sometimes I feel like some people get special treatment. I don’t know if it’s because she’s white, but I feel like there’s a lot of special treatment for different people. I’m not trying to discriminate, but I see that, and I try not to get mad” (50-year-old woman). Referring to specific experiences of anti-Black racism, several Black participants reported that their racialization as Black women contributed to their lack of access to needed shelter and services.

I was trying to get a shelter. It was like, “Oh, you have too much kids. We don’t have no space.” I think it’s a lot to do with the kids because I have four kids . . . So and I think some of them, just based on the way they hear my voice—[they think] that’s just another Black person. (39-year-old-woman)

Participants requested expanded education on IPV and services to address IPV for all gender identities. There was a desire to have increased understanding and societal messaging about IPV as well as increased counseling and shelter access for men: “A woman if she’s in an abusive relationship they’ll move her to a shelter. For men, they don’t offer any of that. What you’re left to do is just try to figure it out yourself or stay in that relationship until you can get out” (49-year-old man).

Men reported that there was a stigma around obtaining services for IPV and that it was “taboo” to talk about experiencing IPV: “Being a male and having to accept the fact that you were the victim in a domestic relationship—I don’t really think that there’s much focus on that. It’s a taboo, and that discourages you from seeking help” (38-year-old man). Some men felt discouraged to disclose IPV because their partner was a man, they were older, or physically larger than their partner: “It’s not just we’re both male. I mean he’s significantly younger than me and smaller than me. So, it looks weird” (53-year-old man).

## Discussion

In a qualitative study of adults experiencing homelessness who reported IPV, we discovered six primary themes regarding IPV and homelessness: (1) IPV led to homelessness and impeded returns to housing; (2) Survivors would remain in abusive relationships as a survival strategy to maintain access to financial resources and housing; (3) Participants reported a complex interaction between alcohol or substance use and IPV, which escalated violence; (4) They requested equitable and expanded access to housing, shelter, social services, and financial support; (5) Survivors described a lack of support for their needs in the healthcare system; and (6) experiences of racial discrimination in multiple systems, including DV shelters and the child welfare system.

Our work aligns with research indicating that IPV puts people at risk of experiencing homelessness (Chan et al., 2021; Pavao et al., 2007). It supports identified mechanisms linking the two, including lack of financial independence (Adams et al., 2012; Anderson & Saunders, 2003; Domestic Violence and Homeless Services Coalition, 2020; Galano et al., 2013; Peled & Krigel, 2016). Our study adds to this literature by showing the ways in which IPV can lead to homelessness for men and women and how it can cause recurrent or prolonged homelessness. We found that property damage, noise complaints, financial coercion, and stalking resulting from IPV led to eviction, poor credit, and a reputation as a bad tenant, making it harder for participants to permanently exit homelessness. Limited societal or community resources played a significant role in compounding these intersecting adverse experiences (Little, 2015).

Permanently leaving an abusive relationship is challenging (Storer et al., 2021). Partners can use financial abuse (Adams et al., 2008; Tarshis, 2022) and children as mechanisms of control to keep survivors in relationships (Clements et al., 2022; Domestic Violence and Homeless Services Coalition, 2020). Although participants were forced to lose housing to leave abusive relationships, they had stayed in harmful relationships longer than desired due to fear that leaving would render them homeless or would be detrimental to their children. They endured IPV to create safety plans for their children and avoid exposing them to homelessness. This work highlights the importance of expanding access to affordable, permanent housing options for IPV survivors to prevent homelessness, reduce exposure to violence, and improve stability for families.

Alcohol and substance use disorders have been associated with increased perpetration of IPV (Crane et al., 2014; R.Yu et al., 2019). Participants reported that intoxication, cravings, and withdrawal symptoms were contributors to violence. They described that intoxication could lead to IPV by triggering traumatic memories and erratic behavior. Similar to previous research findings, we found that methamphetamine intoxication increased violent behavior (Brecht & Herbeck, 2013). Cravings or withdrawal symptoms induce violence through guilt, irritability, and compulsion to get more of the substance. Many people experiencing homelessness are impacted by alcohol or substance use disorders (Kushel et al., 2023; McCarty et al., 1991), which are tied to coping mechanisms for trauma or mental illness (Ferguson et al., 2015; Opalach et al., 2016). Participants reported the substance or alcohol would worsen IPV and lead to homelessness. Embedding alcohol and substance use disorder treatment into IPV intervention programs or policies may be important strategies to respond to violence and prevent homelessness (Gilchrist et al., 2019; Murphy & Ting, 2010; Wilson et al., 2014).

Not receiving support to address IPV directly contributed to homelessness. Previous work supports our findings, with survivors reporting challenges in access to and knowledge of subsidized housing, shelter and social programs for themselves and their children (Domestic Violence and Homeless Services Coalition, 2020; National Network to End Domestic Violence, 2023). Survivors looking to leave abusive partners and avoid homelessness were unable to access DV shelters because they were full and did not have the capacity to admit another person. Those who were able to enter DV shelters reported they were grateful for their privacy and security. However, time-limited DV shelter stays and lack of affordable housing hindered participants’ ability to achieve safety and housing stability (Fraga Rizo et al., 2022; Wood, McGiffert, et al., 2022). Despite the growing unmet need for shelter among survivors, DV programs have decreased shelter programs due to insufficient funding (National Network to End Domestic Violence, 2023). Our research supports securing ongoing funding for increased access to and capacity in DV programs allowing for (1) increased access to permanent affordable housing through dedicated rental subsidies or housing vouchers (Wilkey et al., 2019), (2) flexible funding to address financial needs facing survivors (Decker et al., 2022; Lopez-Zeron et al., 2019; Sullivan et al., 2023), and (3) non-time-limited stays in DV shelters or transitional housing,

Healthcare providers have the capacity to disseminate IPV resources and provide treatment for IPV-related health conditions in a confidential manner (Maquibar et al., 2023; Tavrow et al., 2017; Trabold et al., 2023; US Preventive Services Task Force et al., 2018). However, most participants did not seek help from the healthcare system for their IPV. Many believed providers lacked the knowledge, resources, or empathy to support them. Participants wanted healthcare providers to acknowledge their individual experiences of IPV and have resources to aid with safety planning and expedited entrance to shelter (Kalra et al., 2017). Participants feared disclosing IPV to providers as it could lead to involvement in the criminal legal system or child welfare system and cause worse outcomes for themselves, their partner, or their children (Rancher et al., 2021; Robinson et al., 2021). Mandatory reporting laws for healthcare workers may be a barrier to IPV disclosure and worsen outcomes, particularly for IPV survivors with historically marginalized identities (Lippy et al., 2020). Healthcare settings may better facilitate IPV disclosure and subsequent treatment by optimizing restorative and transformative practices promoting healing and repair from IPV over criminalization (Kim, 2018).

IPV disproportionately impacts people with minoritized identities, particularly Multiracial, Black, and Native people, and LBGTQ+ individuals (Bermea et al., 2021; Leemis et al., 2022; Messinger, 2020). Black and Latina women reported decreased access to DV shelters due to their race or ethnicity and discriminatory practices that favored white survivors or specifically discriminated against Black women. There are reported inequities in access to DV services and housing for survivors of color and LGBTQ+ survivors (Bermea et al., 2021; Brignone & Gomez, 2022). Although our findings align with studies showing more severe IPV among women compared to men (Fanslow et al., 2023), participants highlighted the importance of expanding IPV services to survivors of all gender identities. Men voiced stigma around reporting IPV, decreased access to services, and lack of insight into the dynamics of same-sex relationships (Floyd et al., 2016; Scott-Storey et al., 2023). Centering racial equity, expanding access to services for all gender identities, creating LGBTQ+ affirming spaces, and accounting for the intersectional experiences of survivors are vital to designing programs for people experiencing IPV (Jordan et al., 2020).

## Limitations

Our analysis included any participants who talked about IPV from the seven qualitative sub-studies. For those in the sub-study that focused on the experience of IPV, participant comments may have been influenced by its focus. Most of our cohort identified as a cis-gender woman. We did not purposively oversample LGBTQ+ individuals; however, given the increased risk of IPV among the LGBTQ+ community, further studies are needed to explore the needs of LGBTQ+ survivors. We acknowledge our prior experiences and beliefs influence our research. We incorporated reflexivity throughout our analysis process, created a diverse analytic team with lived expertise in IPV and homelessness, and presented our findings for regular input from advocates, policymakers, and organizations in the field of IPV.

## Implications

Survivors of IPV are at high risk of experiencing homelessness and facing additional barriers to exiting homelessness. To improve the safety and well-being of people who have experienced IPV, we need to create sustainable solutions at the intersection of IPV and homelessness with longitudinal financial support. Programs addressing IPV among people at risk of homelessness need to increase access to permanent affordable housing, flexible funding, non-congregate shelter options, and supportive services that address the needs of survivors specifically. Access to these interventions must be equitable, address racial inequities, and accommodate the needs of LGBTQIA+ individuals.
